# The genome sequence of the Straw Grass-veneer moth, *Agriphila straminella *(Denis & Schiffermüller), 1775

**DOI:** 10.12688/wellcomeopenres.22844.1

**Published:** 2024-08-07

**Authors:** Douglas Boyes, Mark R. Young, University of Oxford and Wytham Woods Genome Acquisition Lab, Darwin Tree of Life Barcoding collective, Wellcome Sanger Institute Tree of Life Management, Samples and Laboratory team, Wellcome Sanger Institute Scientific Operations: Sequencing Operations, Wellcome Sanger Institute Tree of Life Core Informatics team, Tree of Life Core Informatics collective, Darwin Tree of Life Consortium

**Affiliations:** 1 UK Centre for Ecology & Hydrology, Wallingford, England, UK; 2 University of Aberdeen, Aberdeen, Scotland, UK

**Keywords:** Agriphila straminella, Straw Grass-veneer moth, genome sequence, chromosomal, Lepidoptera

## Abstract

We present a genome assembly from an individual male Straw Grass-veneer moth, *Agriphila straminella* (Arthropoda; Insecta; Lepidoptera; Crambidae). The genome sequence has a length of 511.50 megabases. Most of the assembly is scaffolded into 26 chromosomal pseudomolecules, including the Z sex chromosome. The mitochondrial genome has also been assembled and is 15.36 kilobases in length. Gene annotation of this assembly on Ensembl identified 12,087 protein-coding genes.

## Species taxonomy

Eukaryota; Opisthokonta; Metazoa; Eumetazoa; Bilateria; Protostomia; Ecdysozoa; Panarthropoda; Arthropoda; Mandibulata; Pancrustacea; Hexapoda; Insecta; Dicondylia; Pterygota; Neoptera; Endopterygota; Amphiesmenoptera; Lepidoptera; Glossata; Neolepidoptera; Heteroneura; Ditrysia; Obtectomera; Pyraloidea; Crambidae; Crambinae; *Agriphila*; *Agriphila straminella* (Denis & Schiffermüller), 1775 (NCBI:txid572809).

## Background

The genome of the Straw Grass-veneer, *Agriphila straminella*, was sequenced as part of the Darwin Tree of Life Project, a collaborative effort to sequence all named eukaryotic species in the Atlantic Archipelago of Britain and Ireland. Here we present a chromosomally complete genome sequence for *Agriphila straminella*, based on one male specimen from Wytham Woods, Oxfordshire, UK.

## Genome sequence report

**Figure 1.**
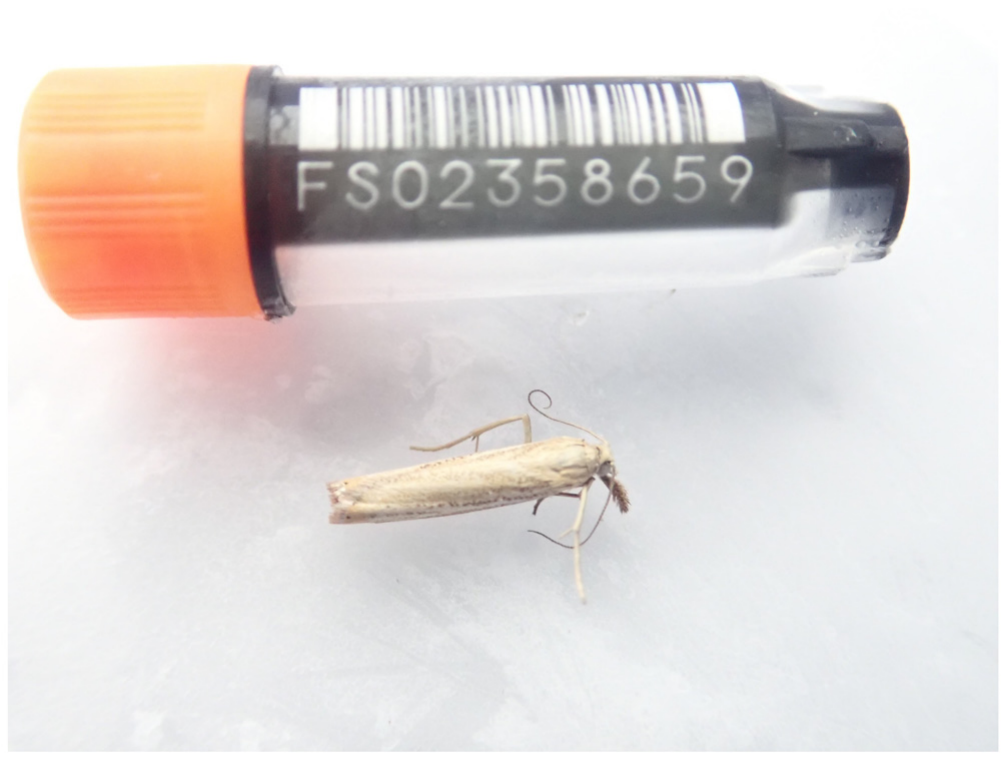
Photograph of the *Agriphila straminella* (ilAgrStra1) specimen used for genome sequencing.

The genome of an adult male *Agriphila straminella* ( Figure 1) was sequenced using Pacific Biosciences single-molecule HiFi long reads, generating a total of 25.79 Gb (gigabases) from 2.03 million reads, providing approximately 47-fold coverage. Primary assembly contigs were scaffolded with chromosome conformation Hi-C data, which produced 96.62 Gbp from 639.89 million reads, yielding an approximate coverage of 189-fold. Specimen and sequencing information is summarised in Table 1.

**Table 1.** Specimen and sequencing data for *Agriphila straminella*.

Project information			
**Study title**	Agriphila straminella (straw grass-veneer)		
**Umbrella BioProject**	PRJEB55944		
**Species**	*Agriphila straminella*		
**BioSample**	SAMEA7701512		
**NCBI taxonomy ID**	572809		
Specimen information			
**Technology**	**ToLID**	**BioSample accession**	**Organism part**
**PacBio long read sequencing**	ilAgrStra1	SAMEA7701694	Whole organism
**Hi-C sequencing**	ilAgrStra2	SAMEA112198564	Head and thorax
**RNA sequencing**	ilAgrStra2	SAMEA112198564	Head and thorax
Sequencing information			
**Platform**	**Run accession**	**Read count**	**Base count (Gb)**
**Hi-C Illumina NovaSeq 6000**	ERR11242506	6.40e+08	96.62
**PacBio Sequel IIe**	ERR10224897	2.03e+06	25.79
**RNA Illumina NovaSeq 6000**	ERR11242507	1.04e+08	15.64

**Figure 2.**
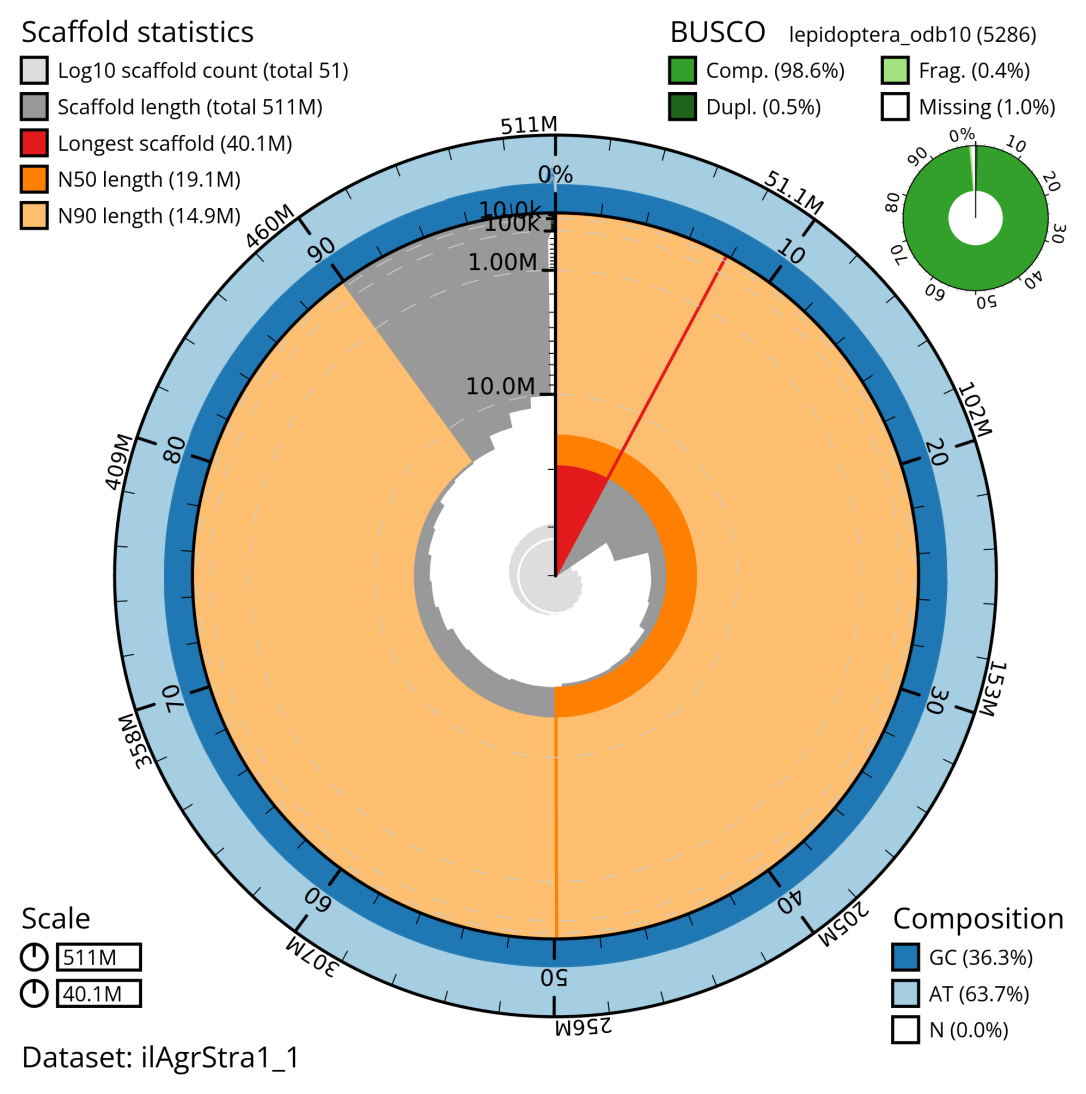
Genome assembly of *Agriphila straminella*, ilAgrStra1.1: metrics. The BlobToolKit snail plot shows N50 metrics and BUSCO gene completeness. The main plot is divided into 1,000 size-ordered bins around the circumference with each bin representing 0.1% of the 511,478,983 bp assembly. The distribution of scaffold lengths is shown in dark grey with the plot radius scaled to the longest scaffold present in the assembly (40,064,077 bp, shown in red). Orange and pale-orange arcs show the N50 and N90 scaffold lengths (19,107,545 and 14,918,624 bp), respectively. The pale grey spiral shows the cumulative scaffold count on a log scale with white scale lines showing successive orders of magnitude. The blue and pale-blue area around the outside of the plot shows the distribution of GC, AT and N percentages in the same bins as the inner plot. A summary of complete, fragmented, duplicated and missing BUSCO genes in the lepidoptera_odb10 set is shown in the top right. An interactive version of this figure is available at https://blobtoolkit.genomehubs.org/view/ilAgrStra1_1/dataset/ilAgrStra1_1/snail.

**Figure 3.**
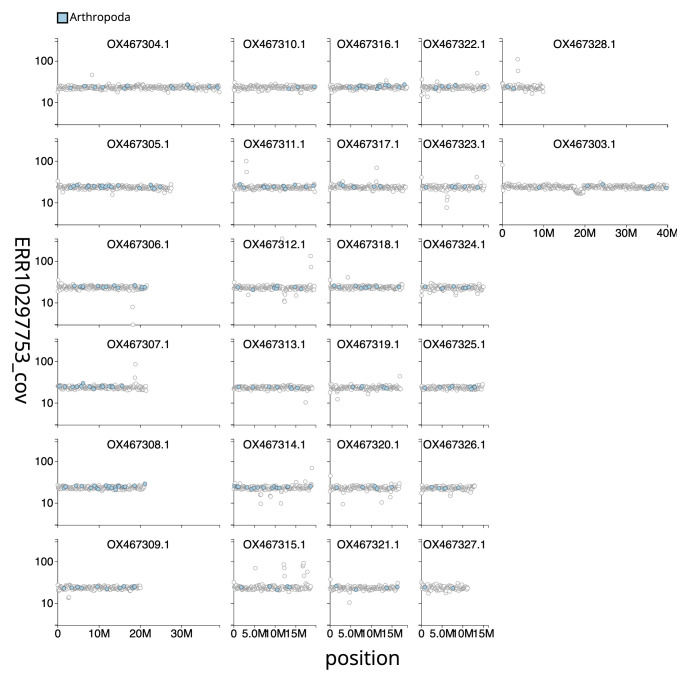
Genome assembly of *Agriphila straminella*, ilAgrStra1.1: in ERR10297753 against position for sequences in the assembly. Windows of 100 kb are coloured by phylum. The assembly has been filtered to exclude sequences with length < 2,550,000.

**Figure 4.**
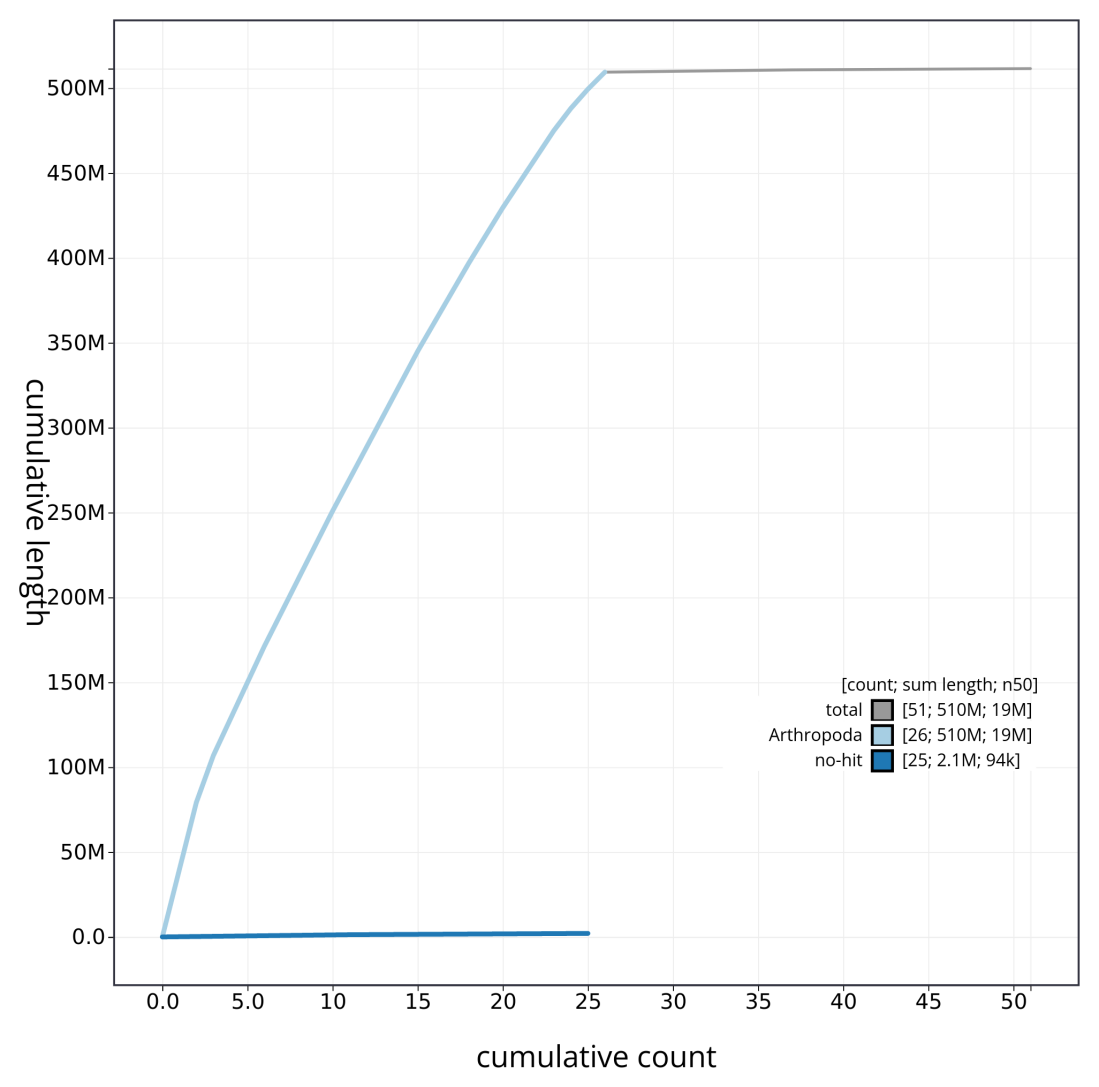
Genome assembly of *Agriphila straminella* ilAgrStra1.1: BlobToolKit cumulative sequence plot. The grey line shows cumulative length for all sequences. Coloured lines show cumulative lengths of sequences assigned to each phylum using the buscogenes taxrule. An interactive version of this figure is available at https://blobtoolkit.genomehubs.org/view/ilAgrStra1_1/dataset/ilAgrStra1_1/cumulative.

**Figure 5.**
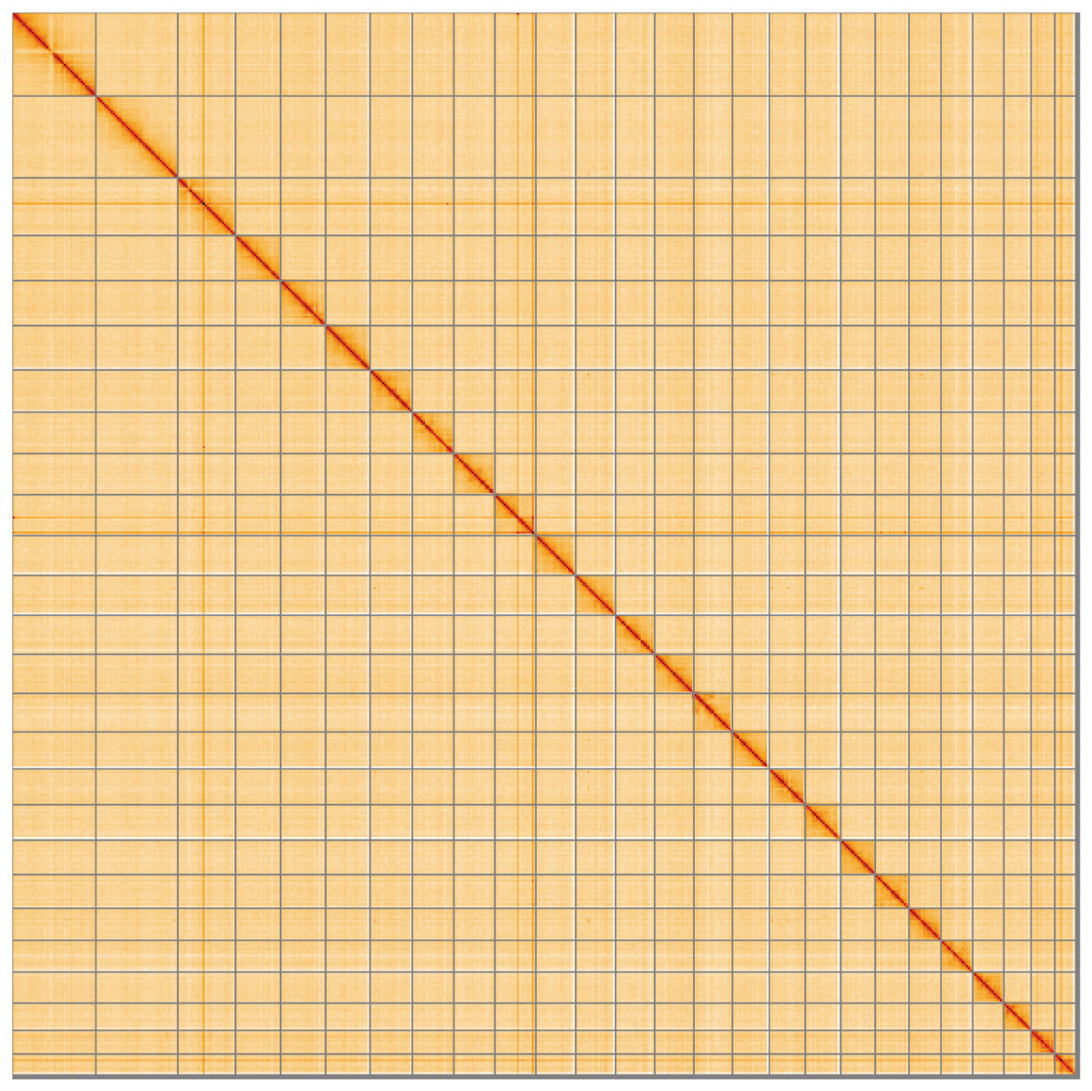
Genome assembly of *Agriphila straminella* ilAgrStra1.1: Hi-C contact map of the ilAgrStra1.1 assembly, visualised using HiGlass. Chromosomes are shown in order of size from left to right and top to bottom. An interactive version of this figure may be viewed at https://genome-note-higlass.tol.sanger.ac.uk/l/?d=ZmCD2N9hTMyG_-iQu0h00w.

Manual assembly curation corrected eight missing joins or mis-joins and two haplotypic duplications, reducing the assembly length by 0.72%, increasing the scaffold number by 2.0%. The final assembly has a total length of 511.50 Mb in 50 sequence scaffolds with a scaffold N50 of 19.1 Mb ( Table 2), with nine gaps. The snail plot in Figure 2 provides a summary of the assembly statistics, while the distribution of base coverage against position per chromosome is shown in Figure 3. The cumulative assembly plot in Figure 4 shows curves for subsets of scaffolds assigned to different phyla. Most (99.59%) of the assembly sequence was assigned to 26 chromosomal-level scaffolds, representing 25 autosomes and the Z sex chromosome. Chromosome-scale scaffolds confirmed by the Hi-C data are named in order of size ( Figure 5; Table 3). Chromosome Z was assigned by synteny to *Agriphila tristella* (GCA_928269145.1) ( Boyes *et al.*, 2022). While not fully phased, the assembly deposited is of one haplotype. Contigs corresponding to the second haplotype have also been deposited. The mitochondrial genome was also assembled and can be found as a contig within the multifasta file of the genome submission.

**Table 2.** Genome assembly data for *Agriphila straminella*, ilAgrStra1.1.

Genome assembly		
Assembly name	ilAgrStra1.1	
Assembly accession	GCA_950108535.1	
*Accession of alternate haplotype*	*GCA_950109475.1*	
Span (Mb)	511.50	
Number of contigs	60	
Contig N50 length (Mb)	18.5	
Number of scaffolds	50	
Scaffold N50 length (Mb)	19.1	
Longest scaffold (Mb)	40.06	
Assembly metrics *		*Benchmark*
Consensus quality (QV)	62.2	*≥ 50*
*k*-mer completeness	100.0%	*≥ 95%*
BUSCO **	C:98.6%[S:98.1%,D:0.5%],F:0.4%, M:1.0%,n:5,286	*C ≥ 95%*
Percentage of assembly mapped to chromosomes	99.59%	*≥ 95%*
Sex chromosomes	Z	*localised homologous pairs*
Organelles	Mitochondrial genome: 15.36 kb	*complete single alleles*
Genome annotation of assembly GCA_950108535.1 at Ensembl		
Number of protein-coding genes	12,087	
Number of non-coding genes	1,805	
Number of gene transcripts	22,391	

* Assembly metric benchmarks are adapted from column VGP-2020 of “Table 1: Proposed standards and metrics for defining genome assembly quality” from Rhie *et al.* (2021). ** BUSCO scores based on the lepidoptera_odb10 BUSCO set using version 5.3.2. C = complete [S = single copy, D = duplicated], F = fragmented, M = missing, n = number of orthologues in comparison. A full set of BUSCO scores is available at https://blobtoolkit.genomehubs.org/view/ilAgrStra1_1/dataset/ilAgrStra1_1/busco.

**Table 3.** Chromosomal pseudomolecules in the genome assembly of *Agriphila straminella*, ilAgrStra1.

INSDC accession	Name	Length (Mb)	GC%
OX467304.1	1	39.22	36.0
OX467305.1	2	27.63	36.5
OX467306.1	3	21.63	36.0
OX467307.1	4	21.56	36.5
OX467308.1	5	21.29	36.5
OX467309.1	6	20.2	36.5
OX467310.1	7	19.89	36.5
OX467311.1	8	19.71	36.5
OX467312.1	9	19.63	36.0
OX467313.1	10	19.11	35.5
OX467314.1	11	19.05	36.0
OX467315.1	12	18.74	36.0
OX467316.1	13	18.73	36.0
OX467317.1	14	18.5	36.0
OX467318.1	15	17.67	36.0
OX467319.1	16	17.21	36.0
OX467320.1	17	16.99	36.0
OX467321.1	18	16.52	36.0
OX467322.1	19	16.18	36.5
OX467323.1	20	15.37	36.5
OX467324.1	21	15.14	37.0
OX467325.1	22	14.92	36.0
OX467326.1	23	13.09	36.0
OX467327.1	24	11.32	37.0
OX467328.1	25	10.06	38.0
OX467303.1	Z	40.06	36.5
OX467329.1	MT	0.02	20.0

The estimated Quality Value (QV) of the final assembly is 62.2 with *k*-mer completeness of 100.0%, and the assembly has a BUSCO v5.3.2 completeness of 98.6% (single = 98.1%, duplicated = 0.5%), using the lepidoptera_odb10 reference set ( *n* = 5,286).

Metadata for specimens, BOLD barcode results, spectra estimates, sequencing runs, contaminants and pre-curation assembly statistics are given at https://links.tol.sanger.ac.uk/species/572809.

## Genome annotation report

The *Agriphila straminella* genome assembly (GCA_950108535.1) was annotated at the European Bioinformatics Institute (EBI) on Ensembl Rapid Release. The resulting annotation includes 22,391 transcribed mRNAs from 12,087 protein-coding and 1,805 non-coding genes ( Table 2; https://rapid.ensembl.org/Agriphila_straminella_GCA_950108535.1/Info/Index). The average transcript length is 17,125.92. There are 1.61 coding transcripts per gene and 7.53 exons per transcript.

## Methods

### Sample acquisition

The specimen used for genome sequencing was an adult *Agriphila straminella* (specimen ID Ox000650, ToLID ilAgrStra1), collected from Wytham Woods, Oxfordshire (biological vice-county Berkshire), UK (latitude 51.77, longitude –1.34) on 2020-07-20 using a light trap. The specimen was collected and identified by Douglas Boyes (University of Oxford) and preserved on dry ice.

The specimen used for Hi-C and RNA sequencing (specimen ID SAN00002602, ToLID ilAgrStra2) was an adult specimen collected by hand from Scougal Moor, Isle of Bute, Scotland, UK (latitude 55.78, longitude –5.05) on 2022-07-02. The specimen was collected and identified by Mark Young (University of Aberdeen) and preserved on dry ice.

The initial species identification was verified by an additional DNA barcoding process according to the framework developed by Twyford *et al*. (2024). A small sample was dissected from the specimens and stored in ethanol, while the remaining parts of the specimen were shipped on dry ice to the Wellcome Sanger Institute (WSI). The tissue was lysed, the COI marker region was amplified by PCR, and amplicons were sequenced and compared to the BOLD database, confirming the species identification ( Crowley *et al.*, 2023). Following whole genome sequence generation, the relevant DNA barcode region was also used alongside the initial barcoding data for sample tracking at the WSI ( Twyford *et al.*, 2024). The standard operating procedures for Darwin Tree of Life barcoding have been deposited on protocols.io ( Beasley *et al.*, 2023).

### Nucleic acid extraction

The workflow for high molecular weight (HMW) DNA extraction at the WSI Tree of Life Core Laboratory includes a sequence of core procedures: sample preparation; sample homogenisation, DNA extraction, fragmentation, and clean-up. In sample preparation, the ilAgrStra1 sample was weighed and dissected on dry ice ( Jay *et al.*, 2023). Tissue from the whole organism was homogenised using a PowerMasher II tissue disruptor ( Denton *et al.*, 2023a). HMW DNA was extracted using the Automated MagAttract v1 protocol ( Sheerin *et al.*, 2023). DNA was sheared into an average fragment size of 12–20 kb in a Megaruptor 3 system with speed setting 30 ( Todorovic *et al.*, 2023). Sheared DNA was purified by solid-phase reversible immobilisation ( Strickland *et al.*, 2023): in brief, the method employs AMPure PB beads to eliminate shorter fragments and concentrate the DNA. The concentration of the sheared and purified DNA was assessed using a Nanodrop spectrophotometer and Qubit Fluorometer using the Qubit dsDNA High Sensitivity Assay kit. Fragment size distribution was evaluated by running the sample on the FemtoPulse system.

RNA was extracted from head and thorax tissue of ilAgrStra2 in the Tree of Life Laboratory at the WSI using the RNA Extraction: Automated MagMax™ *mir*Vana protocol ( do Amaral *et al.*, 2023). The RNA concentration was assessed using a Nanodrop spectrophotometer and a Qubit Fluorometer using the Qubit RNA Broad-Range Assay kit. Analysis of the integrity of the RNA was done using the Agilent RNA 6000 Pico Kit and Eukaryotic Total RNA assay.

Protocols developed by the WSI Tree of Life laboratory are publicly available on protocols.io ( Denton *et al.*, 2023b).

### Sequencing

Pacific Biosciences HiFi circular consensus DNA sequencing libraries were constructed according to the manufacturers’ instructions. Poly(A) RNA-Seq libraries were constructed using the NEB Ultra II RNA Library Prep kit. DNA and RNA sequencing was performed by the Scientific Operations core at the WSI on Pacific Biosciences Sequel IIe (HiFi) and Illumina NovaSeq 6000 (RNA-Seq) instruments. Hi-C data were also generated from remaining head and thorax tissue of ilAgrStra2 using the Arima-HiC v2 kit. The Hi-C sequencing was performed using paired-end sequencing with a read length of 150 bp on the Illumina NovaSeq 6000 instrument.

### Genome assembly, curation and evaluation

***Assembly***

The original assembly of HiFi reads was performed using Hifiasm ( Cheng *et al.*, 2021) with the --primary option. Haplotypic duplications were identified and removed with purge_dups ( Guan *et al.*, 2020). Hi-C reads were further mapped with bwa-mem2 ( Vasimuddin *et al.*, 2019) to the primary contigs, which were further scaffolded using the provided Hi-C data ( Rao *et al.*, 2014) in YaHS ( Zhou *et al.*, 2023) using the --break option. Scaffolded assemblies were evaluated using Gfastats ( Formenti *et al.*, 2022), BUSCO ( Manni *et al.*, 2021) and MERQURY.FK ( Rhie *et al.*, 2020).

The mitochondrial genome was assembled using MitoHiFi ( Uliano-Silva *et al.*, 2023), which runs MitoFinder ( Allio *et al.*, 2020) and uses these annotations to select the final mitochondrial contig and to ensure the general quality of the sequence.

***Assembly curation***

The assembly was decontaminated using the Assembly Screen for Cobionts and Contaminants (ASCC) pipeline (article in preparation). Manual curation was primarily conducted using PretextView ( Harry, 2022), with additional insights provided by JBrowse2 ( Diesh *et al.*, 2023) and HiGlass ( Kerpedjiev *et al.*, 2018). Scaffolds were visually inspected and corrected as described by Howe *et al*. (2021). Any identified contamination, missed joins, and mis-joins were corrected, and duplicate sequences were tagged and removed. The sex chromosome was identified by synteny. The entire process is documented at https://gitlab.com/wtsi-grit/rapid-curation (article in preparation).

***Evaluation of the final assembly***

A Hi-C map for the final assembly was produced using bwa-mem2 ( Vasimuddin *et al.*, 2019) in the Cooler file format ( Abdennur & Mirny, 2020). To assess the assembly metrics, the *k*-mer completeness and QV consensus quality values were calculated in Merqury ( Rhie *et al.*, 2020). This work was done using Nextflow ( Di Tommaso *et al.*, 2017) DSL2 pipelines “sanger-tol/readmapping” ( Surana *et al.*, 2023a) and “sanger-tol/genomenote” ( Surana *et al.*, 2023b). The genome was analysed within the BlobToolKit environment ( Challis *et al.*, 2020) and BUSCO scores ( Manni *et al.*, 2021; Simão *et al.*, 2015) were calculated.

The genome evaluation pipelines were developed using the nf-core tooling ( Ewels *et al.*, 2020), use MultiQC ( Ewels *et al.*, 2016), and make extensive use of the Conda package manager, the Bioconda initiative ( Grüning *et al.*, 2018), the Biocontainers infrastructure ( da Veiga Leprevost *et al.*, 2017), and the Docker ( Merkel, 2014) and Singularity ( Kurtzer *et al.*, 2017) containerisation solutions.

Table 4 contains a list of relevant software tool versions and sources.

**Table 4.** Software tools: versions and sources.

Software tool	Version	Source
BlobToolKit	4.2.1	https://github.com/blobtoolkit/blobtoolkit
BUSCO	5.3.2	https://gitlab.com/ezlab/busco
Hifiasm	0.16.1-r375	https://github.com/chhylp123/hifiasm
HiGlass	1.11.6	https://github.com/higlass/higlass
Merqury	MerquryFK	https://github.com/thegenemyers/MERQURY.FK
MitoHiFi	2	https://github.com/marcelauliano/MitoHiFi
PretextView	0.2	https://github.com/wtsi-hpag/PretextView
purge_dups	1.2.3	https://github.com/dfguan/purge_dups
sanger-tol/genomenote	v1.0	https://github.com/sanger-tol/genomenote
sanger-tol/readmapping	1.1.0	https://github.com/sanger-tol/readmapping/tree/1.1.0
YaHS	yahs-1.1.91eebc2	https://github.com/c-zhou/yahs

### Genome annotation

The Ensembl Genebuild annotation system ( Aken *et al.*, 2016) was used to generate annotation for the *Agriphila straminella* assembly (GCA_950108535.1) in Ensembl Rapid Release at the EBI. Annotation was created primarily through alignment of transcriptomic data to the genome, with gap filling via protein-to-genome alignments of a select set of proteins from UniProt ( UniProt Consortium, 2019).

### Wellcome Sanger Institute – Legal and Governance

The materials that have contributed to this genome note have been supplied by a Darwin Tree of Life Partner. The submission of materials by a Darwin Tree of Life Partner is subject to the **‘Darwin Tree of Life Project Sampling Code of Practice’**, which can be found in full on the Darwin Tree of Life website here. By agreeing with and signing up to the Sampling Code of Practice, the Darwin Tree of Life Partner agrees they will meet the legal and ethical requirements and standards set out within this document in respect of all samples acquired for, and supplied to, the Darwin Tree of Life Project.

Further, the Wellcome Sanger Institute employs a process whereby due diligence is carried out proportionate to the nature of the materials themselves, and the circumstances under which they have been/are to be collected and provided for use. The purpose of this is to address and mitigate any potential legal and/or ethical implications of receipt and use of the materials as part of the research project, and to ensure that in doing so we align with best practice wherever possible. The overarching areas of consideration are:

•   Ethical review of provenance and sourcing of the material

•   Legality of collection, transfer and use (national and international)

Each transfer of samples is further undertaken according to a Research Collaboration Agreement or Material Transfer Agreement entered into by the Darwin Tree of Life Partner, Genome Research Limited (operating as the Wellcome Sanger Institute), and in some circumstances other Darwin Tree of Life collaborators.

## Data Availability

European Nucleotide Archive: Agriphila straminella (straw grass-veneer). Accession number PRJEB55944; https://identifiers.org/ena.embl/PRJEB55944 ( Wellcome Sanger Institute, 2023). The genome sequence is released openly for reuse. The *Agriphila straminella* genome sequencing initiative is part of the Darwin Tree of Life (DToL) project. All raw sequence data and the assembly have been deposited in INSDC databases. Raw data and assembly accession identifiers are reported in Table 1 and Table 2.

## Author information

Members of the University of Oxford and Wytham Woods Genome Acquisition Lab are listed here: https://doi.org/10.5281/zenodo.12157525.

Members of the Darwin Tree of Life Barcoding collective are listed here: https://doi.org/10.5281/zenodo.12158331

Members of the Wellcome Sanger Institute Tree of Life Management, Samples and Laboratory team are listed here: https://doi.org/10.5281/zenodo.12162482.

Members of Wellcome Sanger Institute Scientific Operations: Sequencing Operations are listed here: https://doi.org/10.5281/zenodo.12165051.

Members of the Wellcome Sanger Institute Tree of Life Core Informatics team are listed here: https://doi.org/10.5281/zenodo.12160324.

Members of the Tree of Life Core Informatics collective are listed here: https://doi.org/10.5281/zenodo.5013541.

Members of the Darwin Tree of Life Consortium are listed here: https://doi.org/10.5281/zenodo.4783558.
