# Supplementary material for: The genome sequence of the Straw Grass-veneer moth, Agriphila straminella (Denis & Schiffermüller), 1775
Source: Wellcome Open Res. 2024 Aug 7;9:433. [Version 1] doi: 10.12688/wellcomeopenres.22844.1 (PMC11605175; doi:10.12688/wellcomeopenres.22844.1)
Supplement: Peer review history

# Reviewer report: Reviewer response for version 1

**DOI:** 10.21956/wellcomeopenres.25152.r98218

**Published:** 2024-12-04

**Authors:** Yizhou Chen

**Affiliations:** 1 Elizabeth Macarthur Agricultural Institute, New South Wales Department of Primary Industries Crown Lands Division, Menangle, NSW, Australia

This manuscript reports a high-quality genome assembly for the Straw Grass-veneer moth, *Agriphila straminella*, with a genome size of 511.50 megabases from a single male. It represents a significant contribution to lepidopteran genomics, facilitating studies in ecology, physiology, pollination, and agriculture.

 The manuscript establishes a well-developed protocol from sample collection to high-molecular-weight DNA extraction for long-read sequencing, along with a rigorous pipeline for genome assembly and annotation. I am looking forward to the publication of the Assembly Screen for Cobionts and Contaminants (ASCC) pipeline.

Are sufficient details of methods and materials provided to allow replication by others?

Yes

Is the rationale for creating the dataset(s) clearly described?

Yes

Are the datasets clearly presented in a useable and accessible format?

Yes

Are the protocols appropriate and is the work technically sound?

Yes

Reviewer Expertise:

Animal genomics, genomics of pesticide resistance

I confirm that I have read this submission and believe that I have an appropriate level of expertise to confirm that it is of an acceptable scientific standard.

# Reviewer report: Reviewer response for version 1

**DOI:** 10.21956/wellcomeopenres.25152.r108297

**Published:** 2024-11-28

**Authors:** Théo Léger

**Affiliations:** 1 Leibniz-Institut für Evolutions- und Biodiversitaetsforschung, Museum für Naturkunde Berlin, Berlin, Germany

A well-established protocol has been used to generate this complete genome of Agriphila straminella. An introduction regarding the biology of the moth lacks completely here and should be added. It is unfortunate that the sequenced specimen is a male, because the female sex chromosome W could not be sequenced. This might be something to consider in future sequencing endeavours.

Are sufficient details of methods and materials provided to allow replication by others?

Yes

Is the rationale for creating the dataset(s) clearly described?

Yes

Are the datasets clearly presented in a useable and accessible format?

Yes

Are the protocols appropriate and is the work technically sound?

Yes

Reviewer Expertise:

systematics, phylogeny

I confirm that I have read this submission and believe that I have an appropriate level of expertise to confirm that it is of an acceptable scientific standard.

# Reviewer report: Reviewer response for version 1

**DOI:** 10.21956/wellcomeopenres.25152.r99981

**Published:** 2024-09-30

**Authors:** Bin Zhang

**Affiliations:** 1 China-Australia Joint Institute of Agricultural and Environmental Health, Qingdao Agricultural University, Shenzhen, China

This manuscript provide the chromosomally genome assembly and annotation of Straw Grass-veneer moth, *Agriphila straminella *based on male species. **The mothods using in this manuscript was suitable and results was typically complete. I believe that this high-grade genome sequence would contribute to further explore to this special moth. I recommend to indexing on this journal.

Are sufficient details of methods and materials provided to allow replication by others?

Yes

Is the rationale for creating the dataset(s) clearly described?

Yes

Are the datasets clearly presented in a useable and accessible format?

Yes

Are the protocols appropriate and is the work technically sound?

Yes

Reviewer Expertise:

Insect ecology, bioinformatics, molecular biology

I confirm that I have read this submission and believe that I have an appropriate level of expertise to confirm that it is of an acceptable scientific standard.
